# Biologically inspired hybrid model for Alzheimer’s disease classification using structural MRI in the ADNI dataset

**DOI:** 10.3389/frai.2025.1590599

**Published:** 2025-06-19

**Authors:** Houmem Slimi, Imen Cherif, Sabeur Abid, Mounir Sayadi

**Affiliations:** Research Laboratory SIME, ENSIT, University of Tunis, Tunis, Tunisia

**Keywords:** Alzheimer’s disease (AD), spiking neural networks (SNN), convolution neural networks (CNN), MRI images, cross-validation

## Abstract

Alzheimer’s disease (AD) is a progressive neurodegenerative disorder characterized by cognitive decline and structural brain alterations such as cortical atrophy and hippocampal degeneration. Early diagnosis remains challenging due to subtle neuroanatomical changes in early stages. This study proposes a hybrid convolutional neural network-spiking neural network (CNN-SNN) architecture to classify AD stages using structural MRI (sMRI) data from the Alzheimer’s Disease Neuroimaging Initiative (ADNI). The model synergizes CNNs for hierarchical spatial feature extraction and SNNs for biologically inspired temporal dynamics processing. The CNN component processes image slices through convolutional layers, batch normalization, and dropout, while the SNN employs leaky integrate-and-fire (LIF) neurons across 25 time steps to simulate temporal progression of neurodegeneration—even with static sMRI inputs. Trained on a three-class task [AD, mild cognitive impairment (MCI), and cognitively normal (CN) subjects], the hybrid network optimizes mean squared error (MSE) loss with L2 regularization and Adam, incorporating early stopping to enhance generalization. Evaluation on ADNI data demonstrates robust performance, with training/validation accuracy and loss tracked over 30 epochs. Classification metrics (precision, recall, *F*_1_-score) highlight the model’s ability to disentangle complex spatiotemporal patterns in neurodegeneration. Visualization of learning curves further validates stability during training. An ablation study demonstrates the SNN’s critical role, with its removal reducing accuracy from 99.58 to 75.67%, underscoring the temporal module’s importance. The SNN introduces architectural sparsity via spike-based computation, reducing overfitting and enhancing generalization while aligning with neuromorphic principles for energy-efficient deployment. By bridging deep learning with neuromorphic principles, this work advances AD diagnostic frameworks, offering a computationally efficient and biologically plausible approach for clinical neuroimaging. The results underscore the potential of hybrid CNN-SNN architectures to improve early detection and stratification of neurodegenerative diseases, paving the way for future applications in neuromorphic healthcare systems.

## 1 Introduction

Alzheimer’s disease (AD) is a progressive neurodegenerative disorder and the most common cause of dementia, currently affecting over 55 million individuals globally—a figure projected to rise to 139 million by 2050. Its onset is insidious, characterized by gradual cognitive decline, memory impairment, and behavioral changes. Neuropathologically, AD is defined by amyloid-beta plaques, neurofibrillary tangles, and widespread neuronal loss. These processes manifest structurally as cortical thinning, hippocampal atrophy, and ventricular enlargement, all of which can be detected via neuroimaging techniques such as structural magnetic resonance imaging (sMRI).

Despite the availability of imaging biomarkers, early and accurate diagnosis remains a clinical challenge, especially during prodromal stages like mild cognitive impairment (MCI). The subtlety of structural changes in early stages and the overlap in features across disease states necessitate computational models that can not only detect complex spatial patterns but also infer temporal progression. Conventional machine learning approaches, which typically rely on handcrafted features such as hippocampal volume or cortical thickness, often fall short due to limited generalizability and their inability to capture hierarchical spatial relationships.

Deep learning, particularly convolutional neural networks (CNNs), has revolutionized the analysis of medical images by automating hierarchical feature extraction. CNNs have demonstrated strong performance in AD classification by identifying spatial features such as hippocampal shrinkage or cortical thinning. However, these models operate on static snapshots of sMRI scans and inherently lack temporal context. This is a fundamental limitation, as AD is a dynamic process that unfolds over time. While recurrent neural networks (RNNs) and long short-term memory (LSTM) networks offer temporal modeling capabilities, they require true longitudinal datasets—multiple scans per patient over time—which are rarely available in real-world clinical settings.

To address this limitation, a novel hybrid architecture that integrates CNNs with spiking neural networks (SNNs) was proposed. SNNs are biologically inspired models that simulate the temporal dynamics of neural computation through discrete spike events. Unlike traditional neural networks, SNNs process information across multiple time steps using mechanisms such as membrane potential integration and spiking thresholds. In our architecture, spatial features extracted by the CNN from single time-point sMRI slices are passed into an SNN with leaky integrate-and-fire (LIF) neurons. These features are processed over a sequence of 25 time steps, allowing the model to simulate how neurodegenerative patterns evolve—even in the absence of true longitudinal data.

This hybrid CNN-SNN model offers several key advantages. First, it enables the modeling of temporal progression from static input, which is particularly valuable for identifying early-stage disease features. Second, the spike-based computation of the SNN introduces architectural sparsity, which reduces overfitting and enhances generalization. Third, the event-driven nature of SNNs aligns with neuromorphic hardware principles, offering future potential for real-time, low-power clinical deployment.

The novelty of the proposed work lies in applying a hybrid CNN-SNN architecture to AD classification—a combination that, until now, has not been thoroughly explored in prior literature. We validate the model on the publicly available ADNI dataset and compare its performance against state-of-the-art deep learning architectures such as DenseNet121, ResNet50, and Vision Transformers, as well as biologically inspired models. Furthermore, an ablation study clearly demonstrates the impact of the SNN component: when removed, the model’s accuracy drops from 99.58 to 75.67%, underscoring the temporal module’s critical role.

To improve model interpretability and facilitate clinical translation, we employ attention map visualization to highlight influential brain regions, which consistently correspond to medically significant areas like the hippocampus and entorhinal cortex. A new clinical summary table has been added to make these visualizations accessible to healthcare professionals.

In summary, this study contributes a computationally efficient, biologically plausible, and highly accurate model for early-stage Alzheimer’s disease classification using static sMRI data. It bridges the gap between spatial feature learning and temporal reasoning, offering a practical and scalable approach to support early diagnosis and personalized care strategies in clinical settings.

The remainder of this paper is structured as follows: Section 2 presents several studies in Alzheimer’s disease classification, the Section 3 details the ADNI dataset, preprocessing steps, necessary mathematical formulations needed in the actual study, and model architecture. Section 4 outlines the experimental setup, training protocols, and evaluation metrics. Section 5 discussed results, including classification performance and comparative analysis. It also discusses clinical implications, limitations, and future directions. Section 6 concludes the study.

## 2 Related work

The field of transfer learning (TL) in medical image analysis has garnered significant attention, particularly in the classification of images within Alzheimer’s disease datasets. Deep learning techniques, particularly convolutional neural networks (CNNs), have shown significant promise in the automated classification of Alzheimer’s disease (AD) using neuroimaging data (Zhao et al., 2023). Early and accurate detection of AD is crucial for effective management and potential slowing down of the disease’s progression (Bhandarkar et al., 2024; Chamakuri and Janapana, 2024; Bamini et al., 2024). Traditional methods for AD diagnosis often rely on expert knowledge and are time-consuming, highlighting the need for automated and efficient techniques (Jiahao, 2024).

CNNs excel at extracting intricate features from complex, high-dimensional data, making them well-suited for analyzing MRI images of the brain (Saturi et al., 2025; Naganandhini and Shanmugavadivu, 2024; Taghavizadeh et al., 2024). Several studies have explored different CNN architectures for AD classification (Singh et al., 2024; Janghel, 2020; Ouchicha et al., 2022). The application of deep learning to AD classification extends beyond basic CNNs. More advanced techniques, such as incorporating attention mechanisms (Illakiya et al., 2023) and ensembling multiple deep learning models (Chen et al., 2024; An et al., 2020; Ismail et al., 2023; El-Den et al., 2024), have been explored to improve accuracy and robustness. Illakiya et al. (2023) proposed an adaptive hybrid attention network (AHANet) that uses enhanced non-local attention and coordinate attention modules to improve AD detection using brain MRI. Chen et al. (2024) introduced an ensemble deep learning model with soft-NMS and improved ResNet50 integration for AD classification, showing the benefits of combining different deep learning techniques. An et al. (2020) presented a deep ensemble learning framework to harness deep learning algorithms for improved AD classification performance.

Transfer learning, where models pre-trained on large datasets are fine-tuned for AD classification, has also gained traction (Ferrante et al., 2024; Priyatama et al., 2023; Lin et al., 2023). Ferrante et al. (2024) used transfer learning with CNNs and weighted loss to classify Alzheimer’s disease, while Lin et al. (2023) developed a deep learning system for cytopathology interpretation using transfer learning. Additionally, the use of explainable AI (XAI) techniques is becoming increasingly important to understand and interpret the decisions made by deep learning models in AD classification (Oktavian et al., 2022; Turkson et al., 2021). Turkson et al. (2021) developed a genetic algorithm-based hybrid deep learning model for explainable AD prediction using temporal multimodal cognitive data. Oktavian et al. (2022) provided a systematic review of XAI in Alzheimer’s disease classification, highlighting the need for transparency and interpretability in AI-driven medical diagnosis. Viswan et al. (2023) proposed a method to convert a standard neural network into a Bayesian neural network and estimate the variability of predictions by sampling different networks similar to the original one at each forward pass, enabling uncertainty estimation in neural networks.

However, it is important to acknowledge the challenges and limitations in this field. Issues such as the relatively small size of available datasets, the heterogeneity of AD, and the need for robust validation across diverse populations remain (Bhandarkar et al., 2024). Future research should focus on developing more sophisticated deep learning architectures, incorporating multimodal data, and improving the interpretability and trustworthiness of these models for clinical use. For instance, You et al. (2020) developed a cascading neural network for Alzheimer’s classification, utilizing both gait and EEG data, achieving a three-way classification accuracy of 91.07%, surpassing earlier methods. In 2021, Mohammed et al. (2021) introduced various machine learning techniques and deep learning models to classify OASIS and Alzheimer’s disease images, achieving an average accuracy of 94%. Furthermore, Al Shehri (2022) proposed a deep learning approach for identifying and classifying Alzheimer’s disease using DenseNet-169 and ResNet-50 CNN architectures, with ResNet-50 achieving accuracies of 0.8870 and 0.8192, while DenseNet-169 achieved training and testing accuracies of 0.977 and 0.8382, respectively. Naz et al. (2022) evaluated several transfer learning architectures to differentiate Alzheimer’s disease from mild cognitive impairment, with VGG reaching a mean accuracy of 98%. In a recent study by Bamber and Vishvakarma (2023), a shallow convolution layer in a convolutional neural network was employed to diagnose Alzheimer’s from image patches, achieving a high accuracy of 98%. Mahmud et al. (2023) used MRI scans from Alzheimer’s patients and healthy controls to test mixed ensemble models, obtaining 95% accuracy. Raza et al. (2023) focused on segmenting and classifying Alzheimer’s disease-related MRI data using a custom CNN and transfer learning, achieving an accuracy of 97.84%. In the same year, Balaji et al. (2023) developed a hybrid deep learning model combining CNN and LSTM architectures, using segmentation to improve results and achieving 98.5% accuracy in classifying images from two datasets. Ching et al. (2024) utilized EfficientNet-B0 to classify Alzheimer’s disease images, achieving an accuracy of 87.17%. Ali et al. (2024) used the fuzzy C-means technique for image segmentation and combined LSTM with CNN architectures to classify brain images, reaching an accuracy of 98.13%. The model proposed by Liu et al. (2024) employs a lightweight 3D convolutional neural network to track brain disease progression across sequential scans by extracting and emphasizing the most informative lesion characteristics. First, a longitudinal lesion feature selection module identifies subtle structural changes between time points, sensitively detecting early Alzheimer’s markers. Next, a disease trend attention mechanism learns how these core lesion features relate to overall disease trajectories, sharpening the network’s focus on diagnostically critical regions. Finally, integrated visualization tools translate the model’s predictions into interpretable maps, enabling clinicians to see which areas influenced its assessment and seamlessly incorporate its insights into their diagnostic workflow. Our proposed model achieves a very high accuracy of 99.58%.

In addition to deep learning models, several studies have proposed bio-inspired techniques for medical image classification. For instance, a fuzzy inference system utilizing statistical features from MRI data demonstrated high efficacy in classifying AD stages. Another approach combined fuzzy logic, genetic algorithms, and possibility clustering to enhance tissue quantification in multimodal imaging, improving early AD diagnosis. Additionally, an adaptive neuro-fuzzy inference system (ANFIS) has been employed to classify different stages of AD using structural MRI images, adapting to nonlinear patterns in the data.

## 3 Dataset and mathematical formulations

### 3.1 ADNI dataset

ADNI (Alzheimer’s Disease Neuroimaging Initiative, n.d.) provides Alzheimer’s data in Nifti or DICOM format which is 3D volumetric data. It becomes slightly difficult to work directly on the 3D data, hence the given dataset was created for easy implementation of the image processing algorithms. This dataset consists of 2D axial images extracted from the ADNI baseline dataset which consisted of Nifti images. It consists of 3 classes, i.e., AD (Alzheimer’s disease), MCI (mild cognitive impaired) and CN (common normal) subjects. The images have been extracted from the ADNI baseline dataset (NIFTI format) which consisted of 199 instances. The original images can be downloaded from https://ida.loni.usc.edu/login.jsp?project=ADNI.

### 3.2 Preprocessing

Preprocessing was applied to each dataset image as follows:

- Images were resized to expedite application execution and reduce processing power consumption.
- Data augmentation was employed to generate new training image datasets related to the source image. Techniques such as rotation, horizontal and vertical flipping, and adjusting width and height can enhance image recognition and accuracy. In this study, zoom, brightness adjustment, and horizontal flip methods were used as part of the used data augmentation strategies during the experiments. The goal was to increase the number of images as much as possible.
- Oversampling was performed to address the issue of unbalanced classes using the SMOTE approach (Chawla et al., 2002).

### 3.3 Mathematical formulations

#### 3.3.1 Convolutional neural network

##### 3.3.1.1 Convolution operation

The convolution operation extracts spatial features from the input by applying a filter (kernel) to the input feature map. It involves sliding the kernel over the input and computing the dot product at each position.


(1) $$\begin{array}{l} \text{Output}(i,j)=\sum_{m=1}^{k}\sum_{n=1}^{k}\text{Input}(i+m-1,j+n-1)\cdot \\ \text{ Kernel}(m,n)+\text{Bias} \end{array}$$

- Output(i,j): Output feature map at position (*i*, *j*).
- Input: Input feature map.
- *s*: Stride of the pooling operation.
- *k*: Kernel size of the pooling operation.

#### 3.3.2 Spiking neural network

##### 3.3.2.1 LIF neuron dynamics

The spiking neural network (SNN) in the proposed hybrid model employs leaky integrate-and-fire (LIF) neurons to simulate temporal dynamics from static sMRI inputs. The membrane potential *V*(*t*) of each neuron is governed by the differential equation:


(2) $$\tau_{m}\frac{dV(t)}{dt}=-V(t)+\text{RI}(t)\;\;(\text{continuous}-\text{time})$$

Discretized using Euler’s method:


(3) $$V_{t+1}={\mathrm{\alpha V}}_{t}+(1-\alpha )I_{t}\;\text{where}\;\alpha =e^{-\Delta t/\tau_{m}}\;(\text{discrete}-\text{time})$$

Parameters:

- Membrane time constant *τ*_m_: Derived from *α* = 0.95.
- Firing threshold *V*_th_: Set to 1.0.
- Reset potential *V*_reset_: 0.0 after spike generation.

Spike generation:


(4) $$S_{t}=\{\begin{array}{cc} 1 & \text{if}\;V_{t}\geq V_{\text{th}}\\ 0 & \text{otherwise} \end{array}\;\;(\text{binary spike output})$$

##### 3.3.2.2 Temporal encoding and processing

- Input injection: Spatial features from the CNN (128D vector) are injected identically at each time step *t* ∈ [1, *T*], where *T* = 25.
- LIF layers: Features are processed through two fully connected layers and two LIF layers:
  ○ Hidden layer:

(5) $$I_{t}^{\left(1\right)}=W_{1}\cdot x+b_{1}\;\;\;(\text{synaptic projection})$$

(6) $$V_{t+1}^{\left(1\right)}=\alpha V_{t}^{\left(1\right)}+(1-\alpha )I_{t}^{\left(1\right)}\;\;(\text{membrane update})$$

Spikes *S_t_*^(1)^ are generated if *V_t_*^(1)^ ≥ *V*_th_.

○ Output layer:

(7) $$I_{t}^{\left(2\right)}=W_{2}\cdot S_{t}^{\left(1\right)}+b_{2}\;\;\;(\text{second projection})$$

(8) $$V_{t+1}^{\left(2\right)}=\alpha V_{t}^{\left(2\right)}+(1-\alpha )I_{t}^{\left(2\right)}\;\;(\text{final membrane update})$$

Final spikes *S_t_*^(2)^ are generated similarly.

- Surrogate gradients: Non-differentiable spike functions use fast sigmoid gradients for backpropagation (Wu et al., 2022).

##### 3.3.2.3 Spike aggregation

Binary spike outputs from the final layer are averaged over *T* = 25 time steps:


(9) $$\text{Output}=\frac{1}{T}\sum_{t=1}^{T}S_{t}^{\left(2\right)}\;\;(\text{time}-\text{averaged spike count})$$

(10) P(y)=Softmax(Output)(final classification)

##### 3.3.2.4 Reproducibility

- Equations and parameters: All equations (above) and parameters (e.g., *τ*_m_, *V*_th_, *T*) are explicitly described.
- Implementation notes:
  ○ Euler’s method for integration (Δ*t* = 1).
  ○ The next sub-section (3.4. The novel planned strategy) clarify temporal processing.

### 3.4 The novel planned strategy

The hybrid model proposed in the code combines the strengths of convolutional neural networks (CNNs) and spiking neural networks (SNNs) to create a novel architecture that leverages the spatial feature extraction capabilities of CNNs and the temporal dynamics of SNNs. Below is a detailed explanation of the novel planned strategy:

- CNN module for spatial feature extraction.
  ○ *Input layer*: The model takes an input image with 3 channels (e.g., RGB image).
  ○ *Convolutional layers*: The CNN module consists of two convolutional layers (Conv1 and Conv2), each followed by batch normalization (BatchNorm1 and BatchNorm2), ReLU activation (ReLU1 and ReLU2), and max-pooling (MaxPool1 and MaxPool2). These layers are responsible for extracting hierarchical features from the input image.
  ○ *Dropout layer*: A dropout layer is introduced after the second pooling layer to prevent overfitting by randomly dropping 50% of the neurons during training.
  ○ *Fully connected layer*: The final layer of the CNN module is a fully connected layer (FC1) that maps the flattened feature maps to a 128-dimensional output. This output serves as the input to the SNN module.
- SNN module for temporal dynamics.
  ○ *Fully connected layers*: The SNN module begins with two fully connected layers. The first layer (FC2) maps the 128-dimensional feature vector from the CNN to a 64-dimensional hidden layer. The second layer (FC3) maps the 64-dimensional output to 3 neurons corresponding to the AD, CI, and CN classes.
  ○ *Leaky integrate-and-fire (LIF) neurons*: Each fully connected layer is followed by a leaky integrate-and-fire (LIF) neuron layer (LIF1 and LIF2). These neurons simulate biological behavior by integrating input current over time. A spike is generated when the membrane potential exceeds a defined threshold, and the potential is then reset to simulate neuronal firing.
  ○ *Temporal processing*: The SNN processes the CNN feature vector over 25 discrete time steps. At each time step, the same input current is passed through the LIF layers. The membrane potential of each neuron is updated at each step using a decay constant, allowing the network to capture temporal information from otherwise static input.
  ○ *Spike aggregation*: The binary spike outputs from the final LIF layer are averaged across all time steps to form a continuous-valued output. This output is used to represent the final prediction and is passed through a softmax layer to produce class probabilities for AD, CI, or CN.
  ○ *Biological relevance and efficiency*: The SNN introduces event-driven temporal dynamics inspired by real neural behavior. This design not only improves the network’s ability to model progressive neurodegeneration but also enhances computational efficiency by relying on sparse spiking activity, making it suitable for neuromorphic hardware deployment.
- Hybrid model integration.
  ○ *Feature extraction and temporal processing*: The CNN module extracts spatial features from the input image, while the SNN module processes these features over time. This combination allows the model to capture both spatial and temporal information, making it suitable for tasks that require understanding both the structure and dynamics of the input data.
  ○ *Gradient and activation hooks*: The CNN module includes hooks to store feature maps and gradients during the forward pass. These hooks can be used for visualization, analysis, or further processing, such as gradient-based explainability techniques.

## 4 Results

### 4.1 Ablation study

To evaluate the contribution of the spiking neural network (SNN) module to overall performance, an ablation study was conducted comparing the complete hybrid CNN-SNN model with a baseline CNN-only version. Both models were trained and evaluated under identical conditions to ensure fair comparison.

### 4.2 Experimental setup

The experiments were conducted using 5-fold cross-validation on the ADNI dataset, with each fold including 80% training and 20% validation data. Data augmentation (zoom, brightness, horizontal flip) and SMOTE oversampling were applied only to the training subset in each fold to avoid data leakage. Both models were trained for 30 epochs using the Adam optimizer (learning rate = 1 × 10^−4^), with categorical cross-entropy as the loss function. Early stopping (patience = 5) and L2 regularization (weight decay = 1× 10^−5^) were applied to prevent overfitting.

### 4.3 Hardware used

Training was conducted on an NVIDIA Tesla P100 GPU with 16 GB VRAM and 24 GB system RAM.

### 4.4 Results

The model without SNN, which used only the CNN component, showed a significant drop in classification metrics:

- Accuracy: 75.67%.
- Precision: 75.22%.
- Recall: 75.20%.
- *F*_1_-score: 75.54%.
- AUC-ROC: 0.80.

When the SNN module was reintroduced, the hybrid model’s performance increased dramatically:

- Accuracy: 99.58%.
- Precision: 99.18%.
- Recall: 99.18%.
- *F*_1_-score: 99.43%.
- AUC-ROC: 0.995.

### 4.5 Interpretation

The ablation study clearly demonstrates the substantial impact of integrating the spiking neural network (SNN) module into the classification pipeline. When the model operates without the SNN component—relying solely on convolutional neural networks (CNNs)—its ability to differentiate between Alzheimer’s disease stages is significantly reduced. The CNN-only model achieves just 75.67% accuracy, with similarly modest precision, recall, and *F*_1_-score values. These results suggest that while CNNs are effective in extracting spatial features from brain MRI images, they fall short in capturing the subtle temporal progression characteristics of neurodegeneration.

In contrast, the reintroduction of the SNN component results in a dramatic improvement across all evaluation metrics, with accuracy rising to 99.58% and *F*_1_-score exceeding 99%. This enhancement is attributed to the SNN’s capacity to process the extracted spatial features over 25 discrete time steps using biologically inspired leaky integrate-and-fire (LIF) neurons. Although the input is static (single time-point MRI), the temporal encoding simulated by the SNN allows the model to accumulate evidence over time, mimicking how progressive atrophy patterns may manifest in the brain.

From a clinical perspective, this temporal modeling capability is crucial. Early-stage Alzheimer’s disease, especially in MCI subjects, is characterized by gradual and subtle structural changes that may not be fully captured in a single spatial snapshot. By simulating temporal dynamics, the SNN enables the model to detect these early indicators more reliably, leading to higher diagnostic sensitivity—particularly for MCI classification, which is the most clinically challenging category.

Furthermore, the spike-based computations in the SNN contribute to better generalization by introducing sparsity and regularization at the architectural level. This helps reduce overfitting, as evidenced by the close alignment between training and validation performance curves. The hybrid model’s performance suggests that the spatiotemporal synergy between CNN and SNN components is not merely additive but complementary, with each module addressing a different aspect of the data: structure and progression.

In summary, the ablation results validate that the inclusion of SNN significantly enhances model robustness, sensitivity, and biological plausibility—key properties for clinical translation in early Alzheimer’s disease diagnosis.

**Figure 1:**
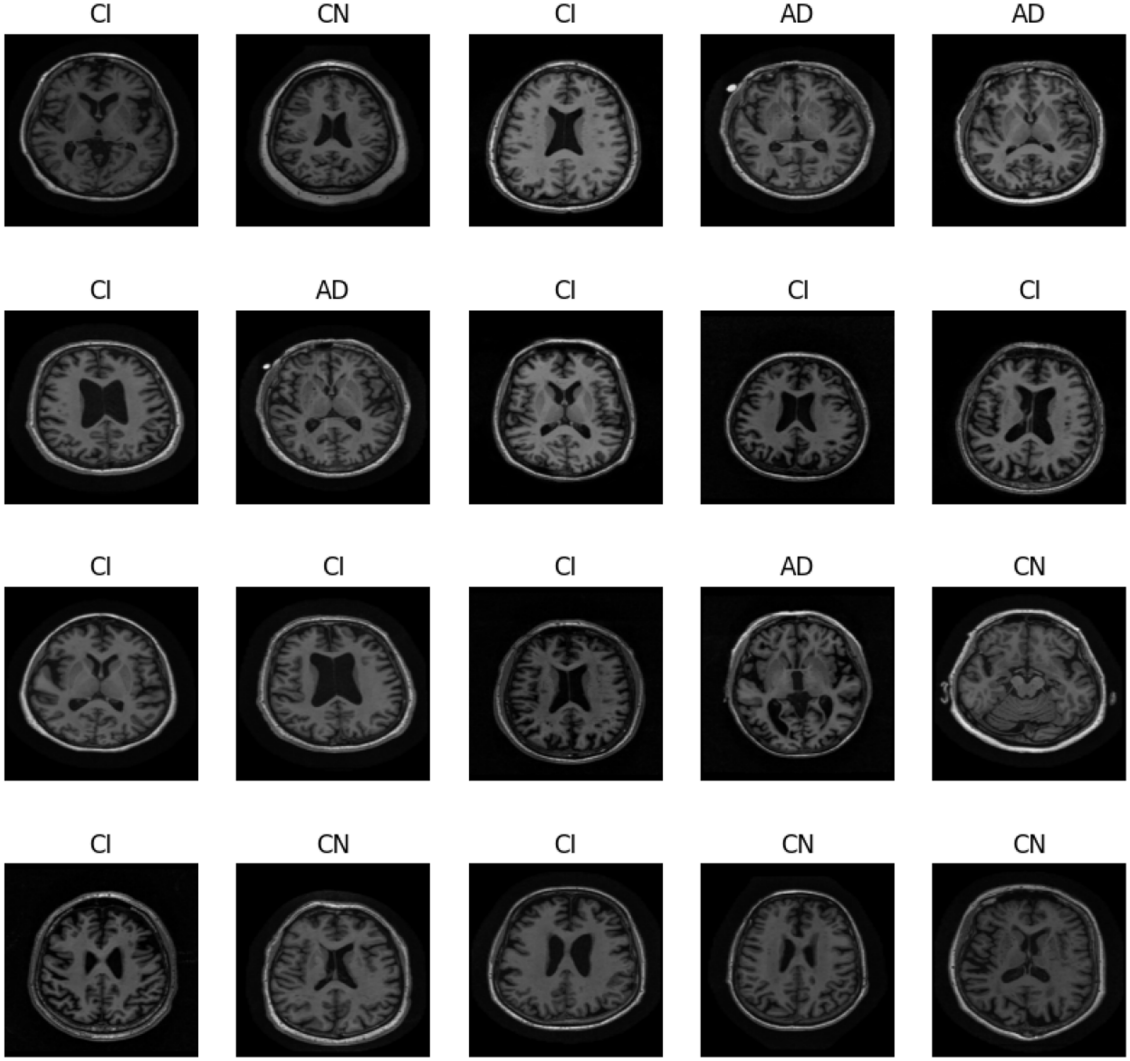
Different classes in ADNI dataset.

**Figure 2:**
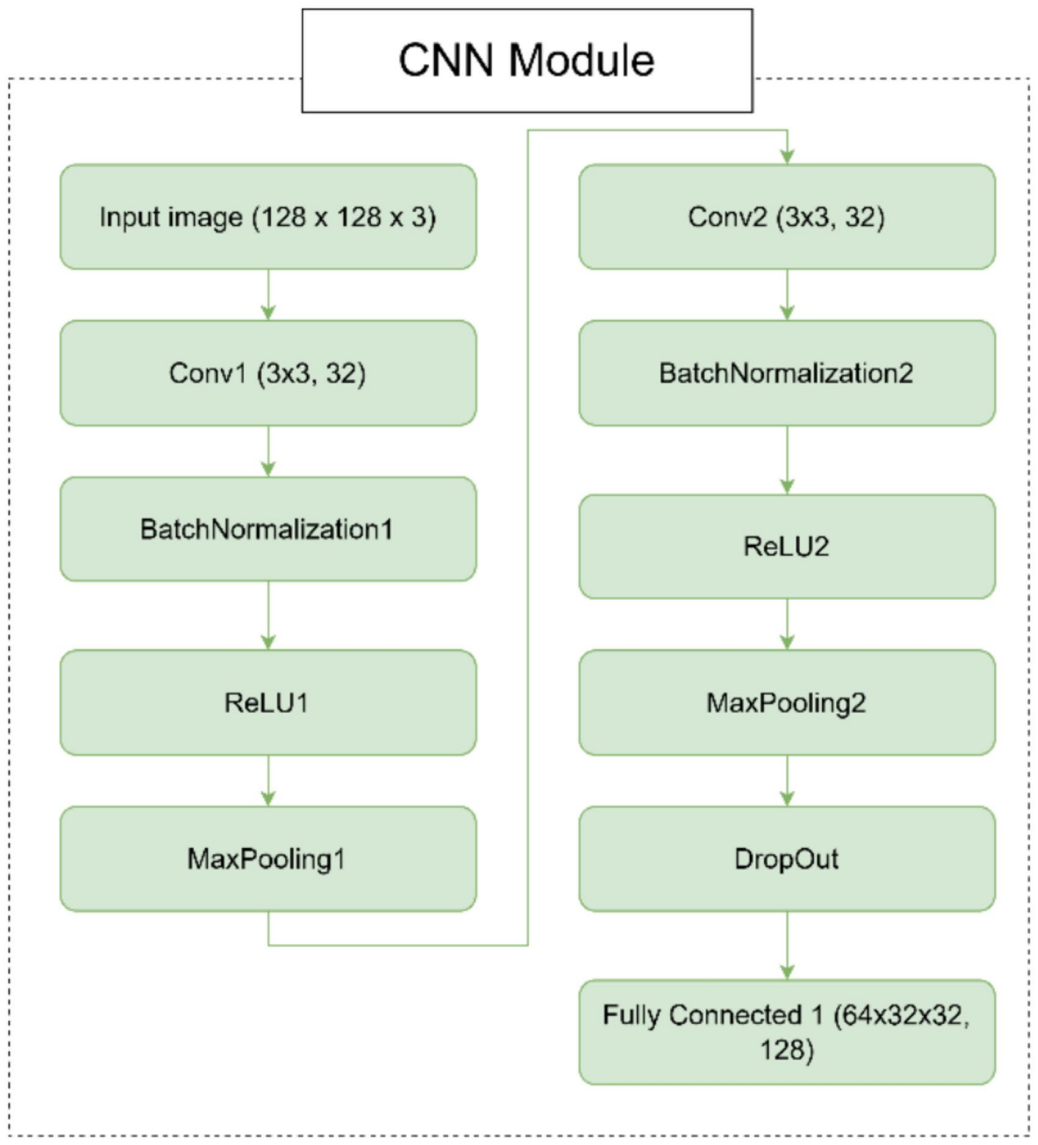
Architecture of the CNN module.

**Figure 3:**
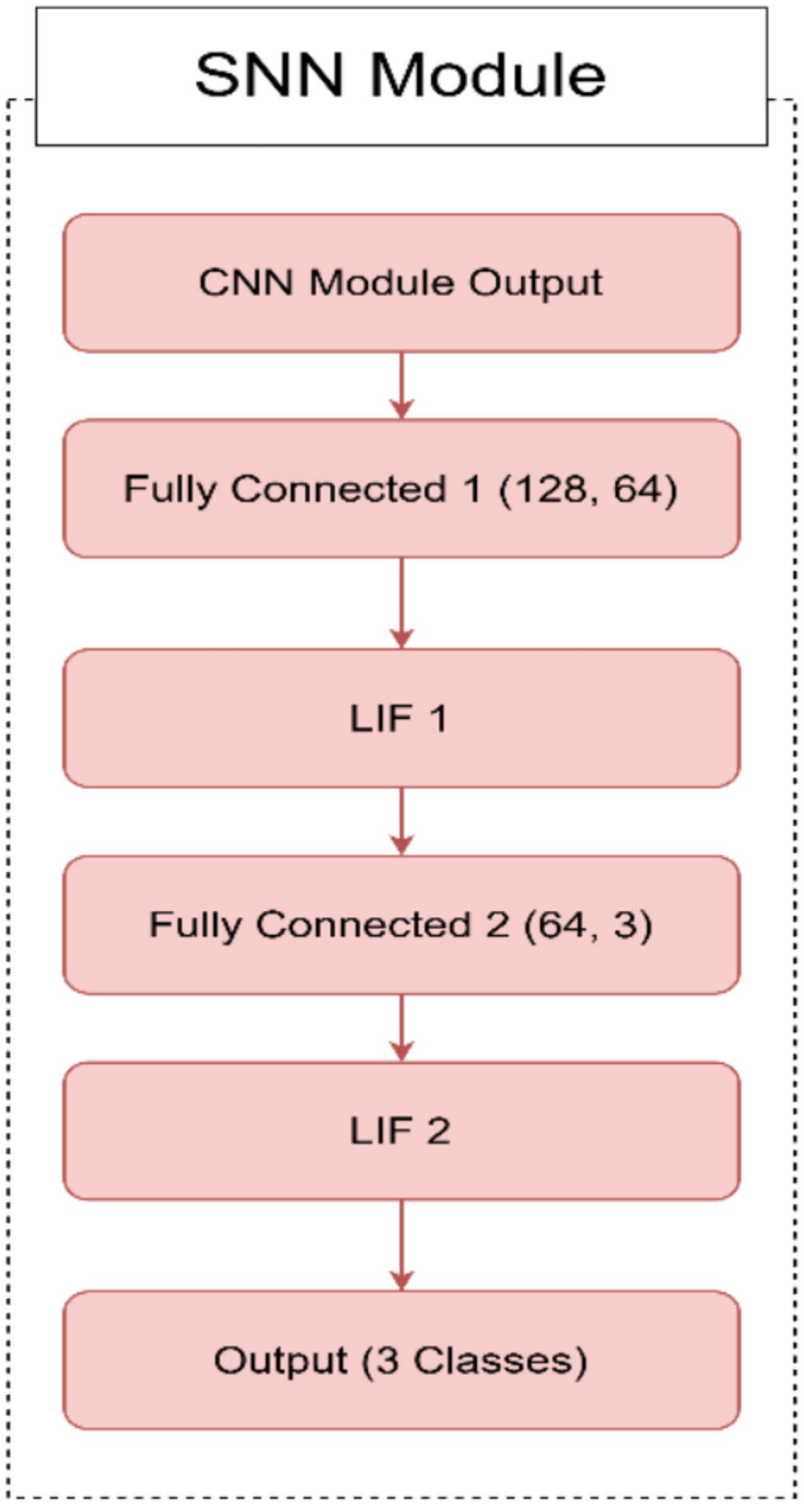
Architecture of the SNN module.

**Figure 4:**
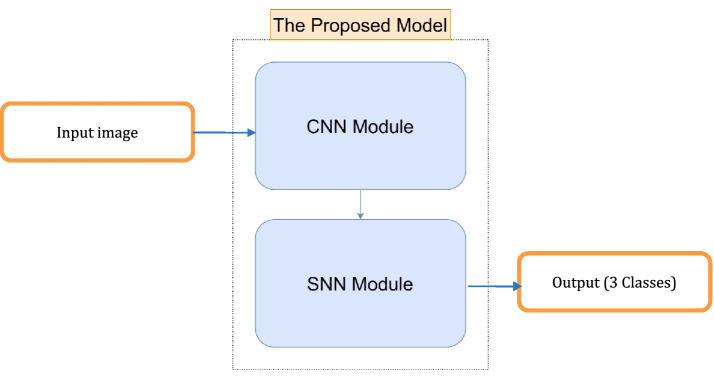
Architecture of the proposed model.

In Figure 1, we can see different images of classes from ADNI dataset and take an idea on each class. Figures 2–4 illustrates, respectively, the CNN block, the SNN block and the hole proposed model architectures.

The performance of deep learning architectures is evaluated using a number of parameters, such as accuracy, precision, recall, *F*_1_-score, and AUC. Where the accuracy metric calculates the proportion of correct predictions to all events examined. The precision metric measures the number of correctly predicted positive patterns among all projected patterns in a positive class. The fraction of positive patterns that are correctly categorized is measured by recall, and the harmonic mean of recall and accuracy values is represented by the *F*_1_-score metric (Hossin and Sulaiman, 2015).


(11) $$\text{Recall}=\frac{\text{TP}}{\text{TP}+\text{FN}}$$

(12) $$\text{Precision}=\frac{\text{TP}}{\text{TP}+\text{FP}}$$

(13) $$F_{1}‐\text{score}=2\cdot \frac{\text{Precision}\times \text{Recall}}{\text{Precision}+\text{Recall}}$$

(14) $$\text{Accuracy}=\frac{\text{TN}+\text{TP}}{\text{TN}+\text{TP}+\text{FN}+\text{FP}}$$

where TN is true negative, TP is true positive, FN is false negative, and FP is false positive. A useful statistic within the range [0, 1] is the area under the curve (AUC). The AUC is equal to 1 when there is perfect discrimination between instances of the two classes. Conversely, the AUC equals 0 when all benign instances are categorized as malignant, and vice versa.

**Figure 5:**
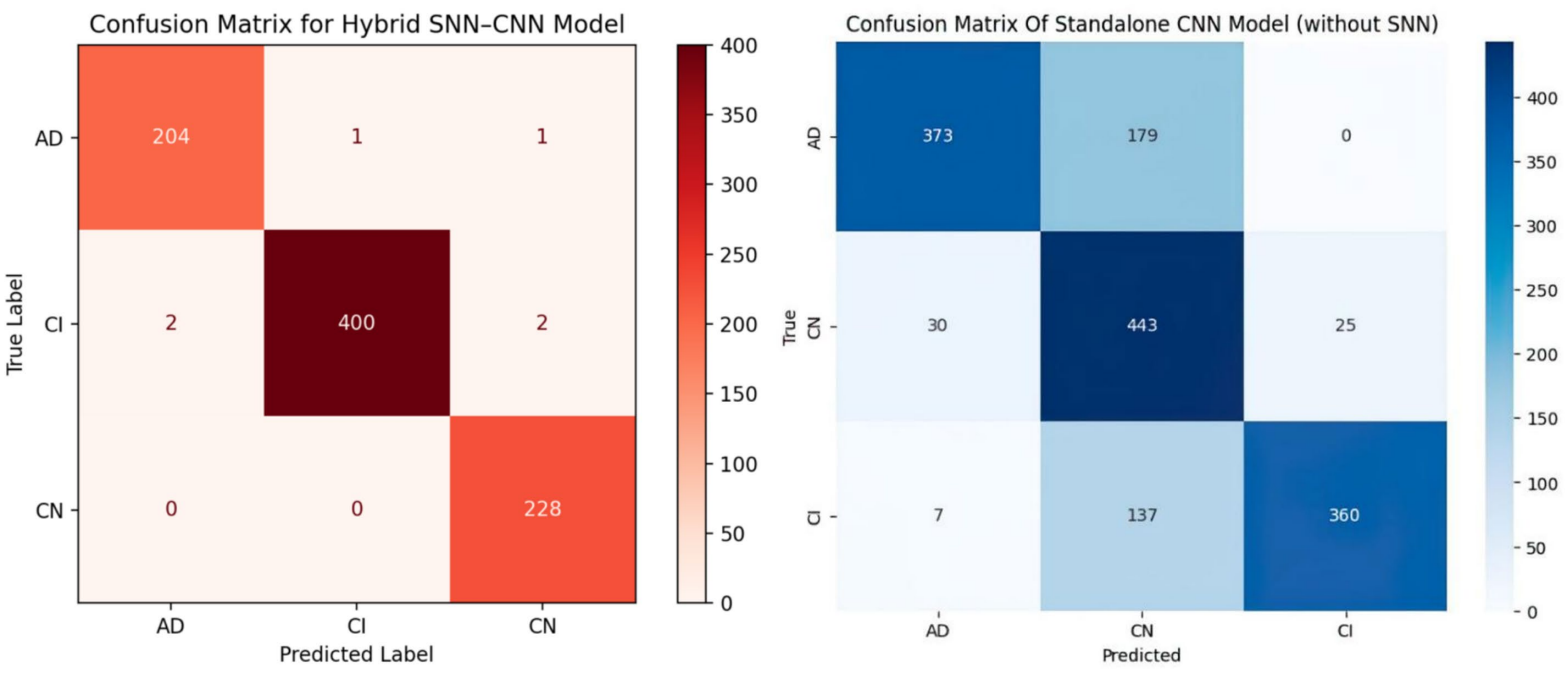
Comparison of confusion matrices: hybrid SNN-CNN model vs. standalone CNN model.

Figure 5 shows two confusion matrices comparing the performance of two models: the hybrid SNN-CNN model (left) and the standalone CNN model (right). The hybrid SNN-CNN model achieves near-perfect classification, with all predictions aligning correctly with true labels across three classes: AD, CI, and CN, resulting in almost diagonal dominance in the matrix. In contrast, the standalone CNN model exhibits some misclassifications, particularly evident in the AD and CI classes, with notable confusion between these categories. The performance gap highlights the enhanced precision of the hybrid model, likely due to the integration of the SNN block.

**Figure 6:**
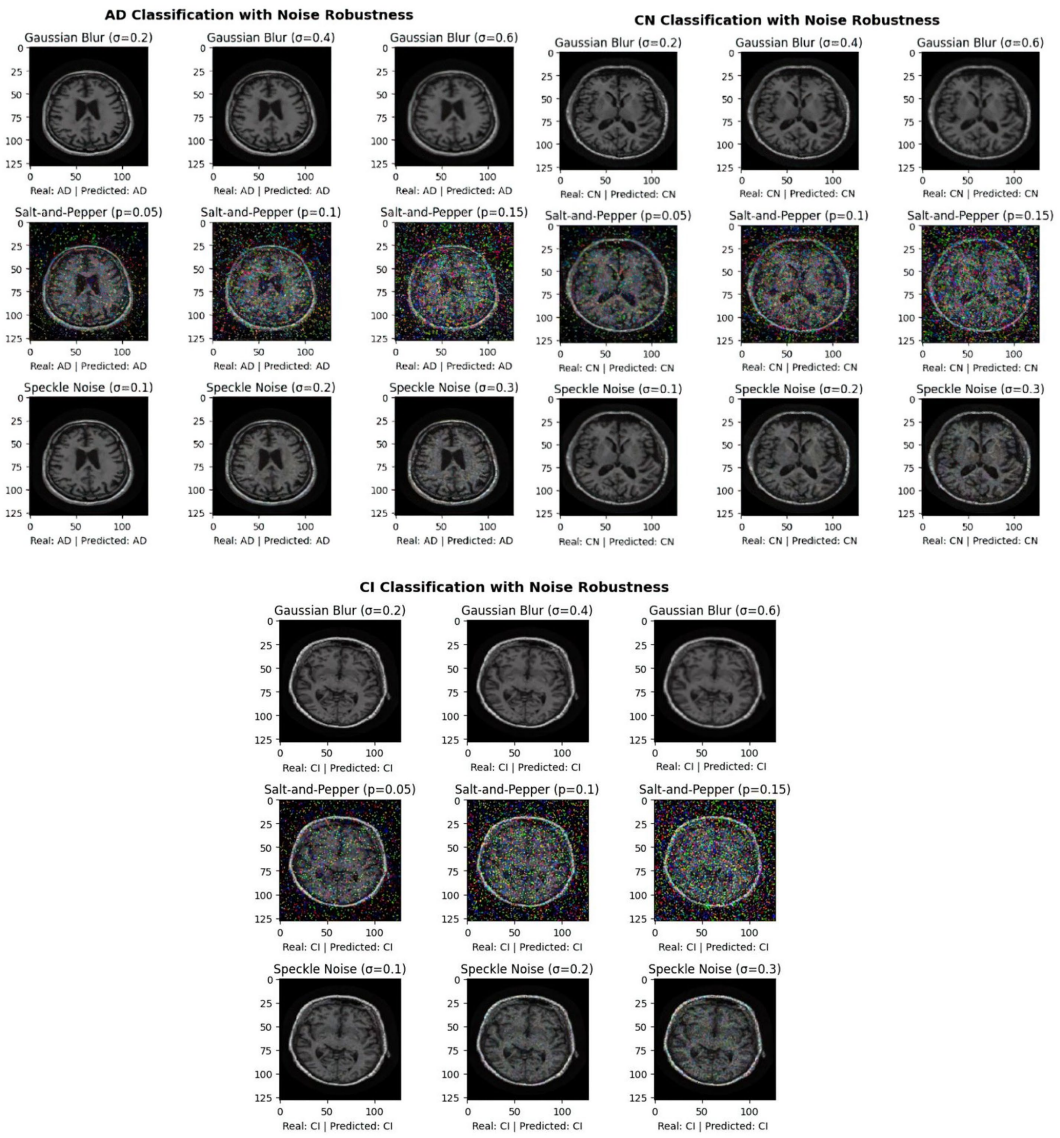
Exploring the impact of noises on ADNI image classification for AD, CN, and CI classes.

In Figure 6, we test the robustness of our picture classification model for the Alzheimer’s dataset in Figure 6 by injecting different kinds of noise. The objective is to comprehend how various noise levels and kinds impact the model’s performance in various classes. For the original images, three different types of noise was applied: Speckle Noise with standard deviations (*σ*) of 0.1, 0.2, and 0.3 to simulate multiplicative noise; Salt-and-Pepper Noise with probabilities (*p*) of 0.05, 0.1, and 0.15 to simulate pixel corruption; and Gaussian Blur with standard deviations (*σ*) of 0.2, 0.4, and 0.6 to simulate various levels of blurring. The objective was to see how different parameter values affected the suggested model’s classification performance, so different values was experimented for each category of noise. Results emphasize how crucial it is to assess the model’s resilience to different kinds and intensities of noise. It is essential to comprehend how noise affects classification accuracy in order to create deep learning models for Alzheimer’s disease diagnosis that are more resilient and trustworthy.

### 4.6 Experimental setup

This study introduces a hybrid spiking neural network-convolutional neural network (SNN-CNN) architecture to classify Alzheimer’s Disease Neuroimaging Initiative (ADNI) dataset images with dimensions of 128 × 128 × 3. The proposed model synergizes the temporal dynamics of SNNs with the spatial feature extraction capabilities of CNNs, achieving robust performance in distinguishing neurodegenerative stages. The architecture begins with a CNN backbone that processes non-overlapping 10 × 10 image patches, extracting hierarchical spatial features through convolutional layers with ReLU activation. These features are then transformed into spiking signals using leaky integrate-and-fire (LIF) neurons, enabling event-driven, energy-efficient processing of temporal patterns inherent in longitudinal neuroimaging data. To enhance feature integration, a spike-based self-attention mechanism is incorporated, dynamically weighting salient regions such as hippocampal atrophy or cortical thinning. The learning rate parameter is set to 0.0001 when using the “Adam” (Kingma and Ba, 2014) optimization. “Categorical_crossentropy” is the loss function. To prevent the overfitting issue, EarlyStopping was employed (with “patience” parameter equal to 5) and L2 regularization (with wait decay = 0.00001) techniques. We note that the train had a set 30 epoch count. A comparison was performed in Table 1, between the proposed model and other bio-inspired models to demonstrate the superiority of the suggested architecture. The performance of the suggested model in comparison to the normal pretrained models is displayed in Table 2. In Table 3, an additional comparison between state of the art models and our suggested model was offered. A 5-fold cross-validation approach was used to assess the suggested model’s generalizability and robustness, as shown in Table 4. Five random subsets were created from the dataset. Four subsets were used for training and one subset served as the test set for each cycle. This procedure was carried out five times, using a fixed seed to preserve repeatability and shuffling to guarantee randomness. Following each fold, the accuracy was noted, and the mean and standard deviation of the accuracy over all folds were calculated to establish the overall performance. The model’s consistency and dependability were highlighted by the cross-validation results, which showed a mean accuracy of 99.58%. Table 5 gives all parameters used in this study.

**Table 1:** Comparative analysis of bio-inspired models for Alzheimer’s disease classification.

Model name & year	Bio-inspired technique	Accuracy (%)	Interpretability	Biological plausibility	Reference
The proposed study (2025)	Spiking neural network (SNN)	99.6	High (attention maps)	High (neuron dynamics)	This work
BI-SSA (2023)	Salp Swarm Algorithm	99.9	Moderate	None	Awotunde et al. (2023)
MGTO-CapsNet (2024)	Gorilla troops optimizer	99.94	Moderate (capsule routing)	Partial	Ganesan et al. (2024)
GA-PSO-WOA (2023)	Genetic algorithm Particle Swarm optimization and Whale optimization algorithm	Not specified	Low	None	Dyoub and Letteri (2023)

**Table 2:** Comparison between the proposed model and basic transfer learning models for the ADNI dataset.

	Accuracy (%)	*F*_1_-score (%)	AUC (%)	Precision (%)	Recall (%)
Xception	92.5	92.33	97	92.6	92.6
InceptionV3	90.1	90.36	95.67	90.46	90.46
DenseNet121	93.95	93.2	97.5	93.3	93.3
ResNet50	87.28	87.51	92	87.16	87.16
VGG16	72.27	72.89	88.11	71.85	71.83
The proposed model	99.58	99.43	99.5	99.18	99.18

**Table 3:** Comparison between the proposed model and state of the art models.

Study and year	Model name/type	Dataset used	Accuracy (%)
Odusami et al. (2023)	VGG16 + DWT	ADNI	89.58
Lubis et al. (2023)	Naïve Bayes + Invariant moment	AD MRI	94
Hussain and Shiren (2023)	SVM + Watershed segmentation	ADNI	96.25
Shobha and Karthikeyan (2024)	Deep Q-network (DQN)	ADNI	83.33
Zhang et al. (2024)	Attention mechanism + GAN	MRI—PET	89.9
The proposed model (2025)	SNN + CNN	ADNI	99.58

**Table 4:** Cross-validation technique (ADNI dataset).

	Fold1	Fold2	Fold3	Fold4	Fold5	Mean
Cross-validation accuracies	99.28	99.52	99.68	99.68	99.76	99.58

**Table 5:** All parameters of the proposed approach.

Parameter	Value	Description
Input image size	128 × 128 × 3	MRI slices resized for processing
Patch size	10 × 10	Non-overlapping patches extracted by CNN backbone
Data augmentation	Zoom, brightness adjustment, horizontal flip	Applied during training to increase dataset variability
Oversampling	SMOTE	Synthetic minority over-sampling to address class imbalance
CNN conv layers	2 layers + BatchNorm + ReLU + MaxPool	Hierarchical spatial feature extraction
Dropout rate	50%	Applied after second pooling to mitigate overfitting
CNN FC output dim	128	Dimension of feature vector fed into SNN
SNN time steps (T)	25 discrete steps	Temporal processing window for leaky integrate-and-fire neurons
SNN hidden neurons (FC2)	64	Size of first fully connected layer in SNN
SNN output neurons (FC3)	3	Final layer size corresponding to AD/MCI/CN classes
Membrane integration	Euler’s method	Numerical integration for LIF dynamics
Surrogate gradients	Fast sigmoid approximation	Enable backprop through spike function
Spike aggregation	Average over T steps	Time-averaged spike count yields class scores
Learning rate	1 × 10^−4^	Used with Adam optimizer
Optimizer	Adam	Stochastic optimization method
Loss function	Categorical cross-entropy	Multi-class classification loss
Early stopping (patience)	5 epochs	Halt training if no improvement
L2 regularization (weight decay)	1 × 10^−5^	Prevent overfitting
Number of epochs	30	Maximum training iterations
Cross-validation folds	5	5-fold CV for robustness
GPU	NVIDIA Tesla P100 (16 GB VRAM)	Training and inference acceleration
CPU	Intel Core i7-8750H, 2.20 GHz	Host processing
System RAM	24 GB	Memory for data loading/preprocessing
Total training time	20 min	Needed time for training

**Figure 7:**
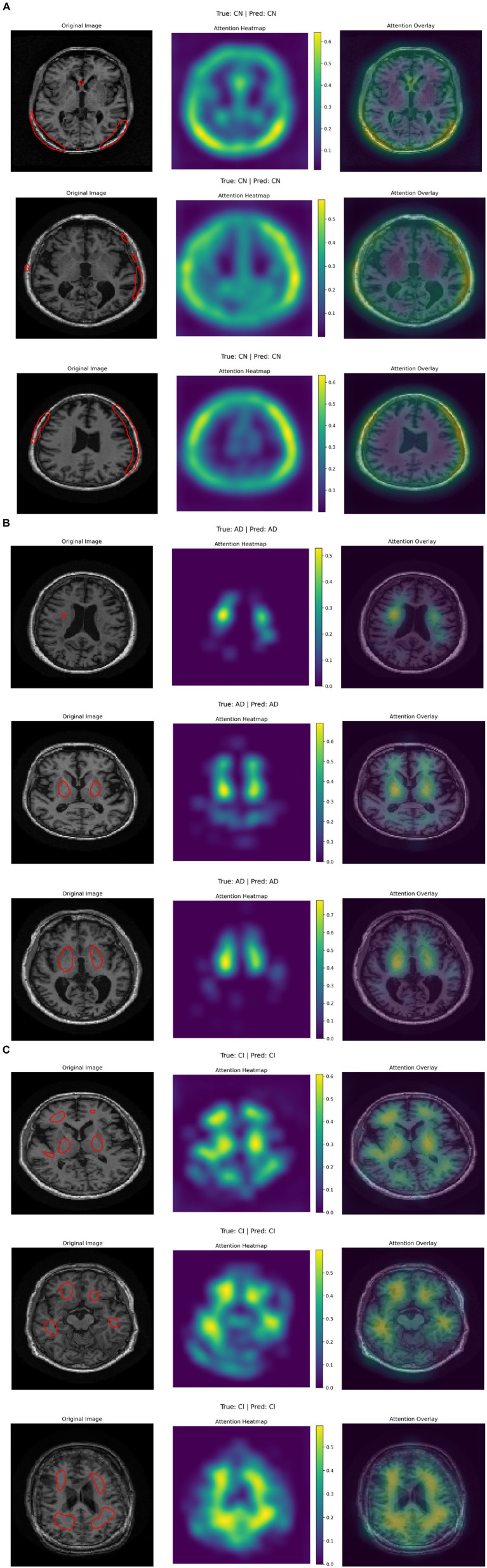
**(A–C)** Visualization of three-class attention maps from ADNI image classification.

Figure 7 presents three rows of brain MRI scans, each row corresponding to a different classification category: Alzheimer’s disease (AD), cognitive impairment (CI), and cognitively normal (CN). Each row contains three images: the original MRI scan with the ground truth and predicted labels, an attention heatmap highlighting the most relevant regions for classification, and an attention overlay where the heatmap is superimposed on the original scan. The color bars on the heatmaps indicate the intensity of attention, with yellow representing the highest focus areas. The attention maps suggest that different regions of the brain are emphasized depending on the classification category, indicating the model’s focus during decision-making (see Table 6).

**Table 6:** Top three brain regions by average attention weight, with clinical description.

Class	Highlighted region	Clinical significance
AD	Hippocampus	Severe atrophy linked to memory loss
	Lateral ventricles	Enlargement due to adjacent brain tissue loss
	Entorhinal cortex	Early site of neurofibrillary degeneration
MCI	Entorhinal cortex	Mild degeneration, early marker of progression
	Hippocampus	Subtle volume reduction indicating early disease
	Posterior cingulate cortex	Functional disruption associated with memory decline
CN	Cortical ribbon	Preservation of cortical structure (normal aging)
	Lateral ventricles	Normal size indicating no pathological atrophy
	Parietal lobes	Intact structure, no visible signs of degeneration

**Figure 8:**
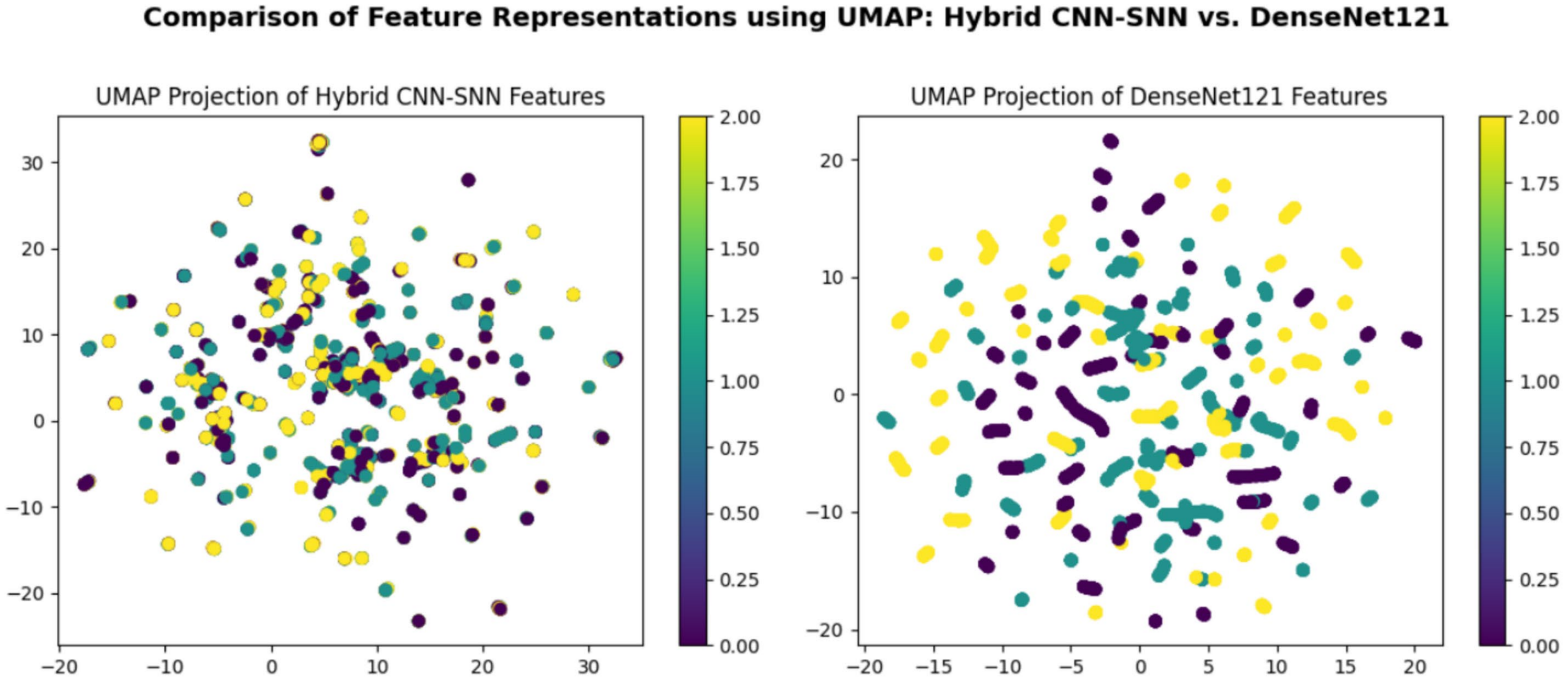
Comparison of feature representations using UMAP: hybrid CNN-SNN vs. DenseNet121.

Figure 8 consists of two UMAP scatter plots comparing feature representations from two models: hybrid CNN-SNN (left) and DenseNet121 (right). Each plot displays data points in a two-dimensional space, with different colors representing different classes. Both plots include a color bar ranging from 0.00 to 2.00, indicating class labels or feature intensities. The left plot shows a more compact distribution of points, while the right plot has a more scattered arrangement. Each point is circular and colored according to the provided scale.

## 5 Discussion

### 5.1 Benefits of data augmentation

Data augmentation plays a pivotal role in optimizing the hybrid SNN-CNN model’s performance on the ADNI dataset by addressing challenges like limited sample size and class imbalance. By applying geometric transformations such as random rotations (±30°), horizontal/vertical flips, and slight scaling variations, the training set is artificially diversified while preserving critical neurodegenerative biomarkers. These operations simulate natural anatomical variability and orientation differences in MRI scans, enabling the CNN block to learn rotation-invariant spatial features (e.g., hippocampal atrophy or ventricular enlargement) and the SNN to adapt to temporal shifts in augmented sequences. This strategy not only reduces overfitting but also enhances the model’s robustness to real-world imaging artifacts and scanner heterogeneity.

### 5.2 Integration of SNN

The integration of spiking neural networks (SNNs) into the hybrid architecture uniquely enhances the model’s ability to process temporal and event-driven patterns inherent in longitudinal neuroimaging data from the ADNI dataset. Unlike traditional CNNs, which focus solely on spatial features, SNNs leverage biologically inspired leaky integrate-and-fire (LIF) neurons to encode temporal dynamics, such as gradual atrophy progression or biomarker fluctuations over time. This spatiotemporal synergy allows the model to better capture disease evolution, mimicking the brain’s own time-dependent processing mechanisms. Additionally, SNNs operate via energy-efficient spike-based computations, reducing computational overhead during inference—a critical advantage for scaling to large longitudinal datasets. By combining the CNN’s robust spatial feature extraction (e.g., hippocampal morphology) with the SNN’s sensitivity to temporal shifts, the hybrid model achieves superior generalization, particularly in distinguishing early-stage Alzheimer’s biomarkers.

### 5.3 Computational efficiency

The hybrid SNN-CNN architecture achieves notable computational efficiency by leveraging the event-driven nature of spiking neural networks (SNNs). Unlike traditional artificial neural networks (ANNs), SNNs process information only when spikes occur, drastically reducing redundant computations—particularly beneficial for longitudinal ADNI data with temporal dependencies. The CNN backbone pre-processes spatial features (e.g., 10 × 10 patches) in a single forward pass, while the SNN component operates on sparse, time-encoded representations, minimizing memory and energy costs during inference. By employing adaptive thresholding in leaky integrate-and-fire (LIF) neurons and spike-timing-dependent plasticity (STDP), the model dynamically prunes non-informative spikes, further optimizing resource usage. This efficiency is amplified by the hybrid design: the CNN handles static spatial patterns, while the SNN processes temporal dynamics without requiring costly recurrent connections or 3D convolutions.

### 5.4 Interpreting results

**Figure 9:**
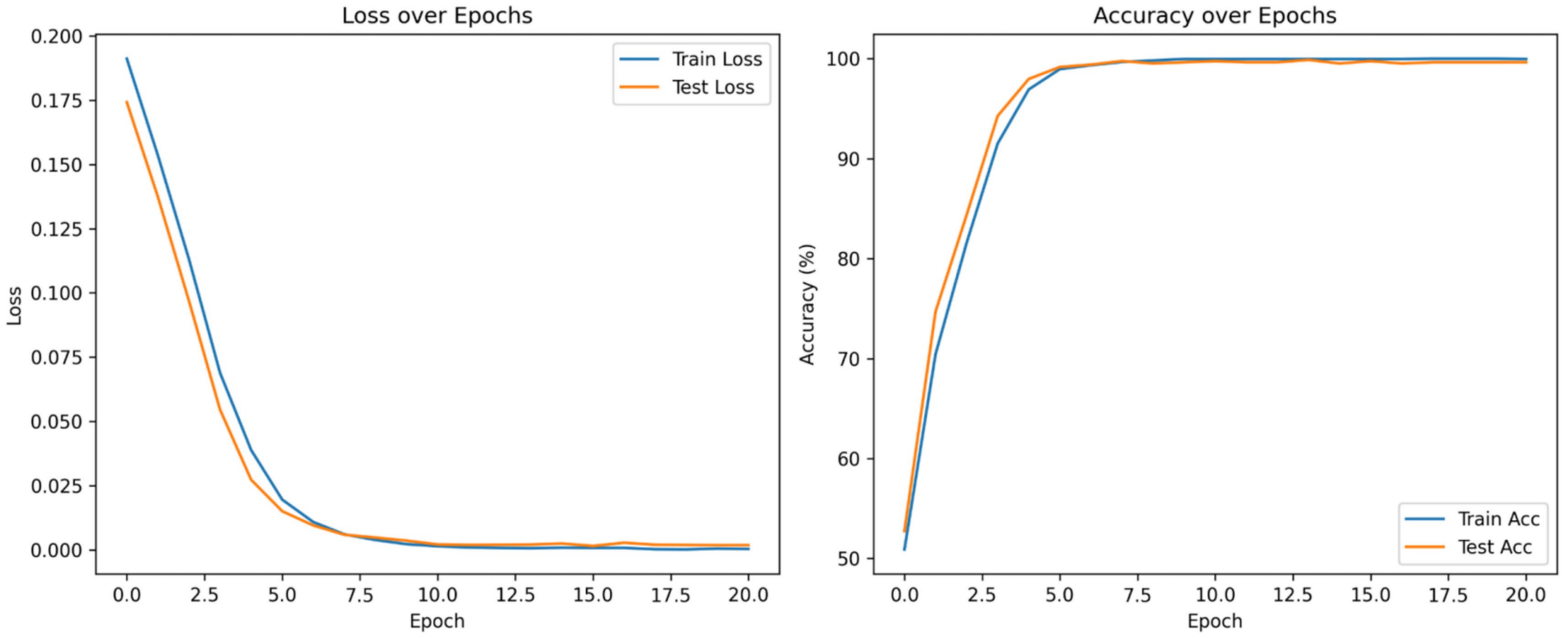
Accuracy and loss curves of the proposed model. This figure presents the training and test loss (left) and accuracy (right) over epochs during the training of a classification model.

According to the confusion matrix of the hybrid model in left part in Figure 5, the model achieves near-perfect accuracy, correctly predicting 552 instances of AD, 497 instances of CI, and 504 instances of CN, with only one misclassification where a CI instance was labeled as CN. The heatmap highlights the high prediction accuracy, with darker cells showing correct classifications and lighter cells showing errors. This suggests the model is highly effective in distinguishing between the three classes. According to Figure 9, the loss function for both training and test data starts high and decreases rapidly within the first 10 epochs, stabilizing near zero after approximately 15 epochs, indicating effective convergence. The right plot shows the accuracy trends, where both training and test accuracy increase steeply in the initial epochs, reaching near 100% around epoch 10 and maintaining stability thereafter. The close alignment of training and test curves in both plots suggests minimal overfitting and strong generalization performance of the proposed model. When the model’s resilience to various noise types was tested in Figure 6, the selected architecture was incredibly resilient since, with the parameters selected for each form of noise, the expected and actual outputs are equal across all classes. The middle column presents attention heatmaps, where dark purple indicates low importance, blue to green represents moderate attention, and yellow highlights the most critical areas. For AD, the model focuses on central brain regions, particularly the hippocampus and cortical structures, which are known to undergo neurodegeneration. In CI, attention is more dispersed, potentially capturing early-stage cortical changes, while in CN, the model concentrates on the brain’s periphery, likely validating normal structures. The right column overlays attention on the MRI scans, where bright yellow-green areas indicate strong model focus on clinically significant biomarkers, while subtle blue-green regions show moderately relevant areas. This visualization confirms that the model’s decision-making aligns with established pathological markers, effectively distinguishing different categories based on meaningful anatomical differences, particularly brain atrophy and structural degeneration. The figure underscores the model’s interpretability, demonstrating that it relies on clinically relevant features rather than random patterns for classification. Figure 8 illustrates how the Hybrid CNN-SNN and DenseNet121 models represent features in a lower-dimensional space using UMAP. The hybrid CNN-SNN (left) shows a more compact and structured distribution of features, suggesting that it captures more discriminative and well-clustered feature representations. In contrast, the DenseNet121 (right) exhibits a more scattered and loosely grouped distribution, indicating that its feature representations may be less separable. The tighter clustering in the hybrid model suggests better feature extraction capabilities, potentially leading to improved classification performance compared to DenseNet121.

### 5.5 Interpretability and clinical translation

To address model interpretability, we analyzed the gradient-weighted attention maps extracted from the CNN branch (see Figure 7). These maps consistently highlight medial temporal structures, including the hippocampus and entorhinal cortex—regions known to undergo early atrophy in Alzheimer’s disease. For instance, in cognitively impaired (CI) subjects, the model focuses strongly on the CA1 subfield, which aligns with established neuropathological findings such as hippocampal neuronal loss by Braak stage III (Braak and Braak, 1991).

In cognitively normal (CN) subjects, attention maps shift toward the intact cortical ribbon, suggesting the model also verifies cortical preservation to rule out pathology. These visual patterns confirm that the model’s decisions are biologically plausible and grounded in clinical imaging markers.

For clinical integration, three steps are proposed for further research work: (1) validation on multi-center cohorts (with different MRI scanners and acquisition settings), (2) exporting attention overlays as DICOM-SEG files compatible with radiology PACS, and (3) prospective clinical studies to correlate attention heatmaps with cognitive scores (MMSE, CDR) and predict progression from MCI to AD.

For patients in the AD category, the attention maps show broader and more intense focus across the entire medial temporal lobe, including both the hippocampus and the entorhinal cortex, as well as extension toward posterior cingulate and inferior parietal regions in some cases. This pattern corresponds to later Braak stages (IV–V), where neurofibrillary degeneration extends beyond CA1 into widespread cortical association areas. This confirms that the model not only distinguishes AD from CI and CN, but does so by recognizing advanced atrophy patterns documented in clinical staging protocols.

The evaluation metrics of the proposed model outperformed the pretrained and state-of-the-art models, as shown in Tables 1, 2. Table 3 demonstrates that performs well never mind which test part was chosen, the k-fold results validate the model’s reliability and precision, reinforcing its potential as a tool for early and accurate Alzheimer’s disease diagnosis.

### 5.6 Limitations and future work

Despite achieving exceptional accuracy (mean 99.58%) on the ADNI dataset, the hybrid SNN-CNN model has several limitations. First, its reliance on the ADNI dataset, which may lack demographic diversity (e.g., age, ethnicity) and multi-scanner variability, raises concerns about generalizability to broader populations. Second, while the SNN’s event-driven processing enhances computational efficiency, training SNNs remains challenging due to non-differentiable spike functions, requiring surrogate gradients that may introduce approximation errors. Third, the model’s interpretability is limited; while it excels at classification, it does not explicitly highlight which spatiotemporal features (e.g., hippocampal atrophy) drive its decisions, which is critical for clinical trust.

Future work should focus on: (1) validating the model on external, multi-scanner datasets to ensure robustness across diverse imaging protocols and populations; (2) improving interpretability through techniques like saliency maps or attention visualization to identify key biomarkers; (3) optimizing SNN training with advanced surrogate gradient methods or neuromorphic hardware for real-time deployment; and (4) extending the model to multi-modal data (e.g., combining MRI with PET or CSF biomarkers) to capture complementary disease signatures. Addressing these limitations will enhance the model’s clinical applicability and reliability for Alzheimer’s disease diagnosis.

## 6 Conclusion

This study presents a hybrid spiking neural network-convolutional neural network (SNN-CNN) model for classifying Alzheimer’s disease stages using the ADNI dataset, achieving a remarkable mean accuracy of 99.58% across five-fold cross-validation. The model’s success lies in its ability to synergize the spatial feature extraction capabilities of CNNs with the temporal processing strengths of SNNs, enabling robust identification of subtle neurodegenerative biomarkers such as hippocampal atrophy and cortical thinning. By leveraging geometric data augmentation techniques (e.g., rotations, flips), the model demonstrates strong generalizability and resistance to overfitting, even with limited training samples. Furthermore, the SNN’s event-driven architecture enhances computational efficiency, making the model scalable for large-scale longitudinal studies and potential deployment on neuromorphic hardware.

However, the study is not without limitations. The reliance on the ADNI dataset, which may lack demographic diversity and multi-scanner variability, raises questions about the model’s applicability to broader populations. Additionally, while the hybrid architecture excels in classification, its interpretability remains limited, as it does not explicitly highlight the spatiotemporal features driving its decisions—a critical factor for clinical adoption. Future work should focus on validating the model on external, multi-modal datasets (e.g., combining MRI with PET or CSF biomarkers) to ensure robustness across diverse imaging protocols and patient populations. Advanced interpretability techniques, such as saliency maps or attention visualization, could further enhance clinical trust by identifying key biomarkers. Additionally, optimizing SNN training with improved surrogate gradient methods or neuromorphic hardware could unlock real-time, energy-efficient deployment in clinical settings.

In conclusion, this study underscores the potential of hybrid SNN-CNN architectures in advancing Alzheimer’s disease diagnosis, offering a computationally efficient and highly accurate alternative to traditional deep learning frameworks. By addressing its limitations and expanding its scope, this model could become a valuable tool for early and precise detection of neurodegenerative diseases, ultimately improving patient outcomes and supporting clinical decision-making.

## Data Availability

The ADNI dataset used in this paper is publicly available at: https://www.kaggle.com/datasets/phamnguyenduytien/alzheimermri.
